# Nitrogen fertilizer reduction in combination with *Azolla* cover for reducing ammonia volatilization and improving nitrogen use efficiency of rice

**DOI:** 10.7717/peerj.11077

**Published:** 2021-03-16

**Authors:** Guoying Yang, Hongting Ji, Hongjiang Liu, Yanfang Feng, Yuefang Zhang, Liugen Chen, Zhi Guo

**Affiliations:** 1 Institute of Agricultural Resources and Environment, Jiangsu Academy of Agricultural Sciences, Nanjing, Jiangsu, China; 2 Key Laboratory for Crop and Animal Integrated Farming, Ministry of Agriculture, Nanjing, Jiangsu, China; 3 Nanjing Institute of Agricultural Sciences in Jiangsu Hilly Area, Jiangsu Academy of Agricultural Sciences, Nanjing, Jiangsu, China

**Keywords:** Nitrogen fertilizer reduction, Azolla cover, Ammonia volatilization, Nitrogen use efficiency, Rice

## Abstract

**Background:**

Excessive nitrogen (N) application rate with low N use efficiency (NUE) caused a considerable amount of N losses, especially ammonia volatilization (AV). Proper N fertilizer reduction (RN) could significantly reduce AV. However, continuous RN led to a nutrient deficiency in the soil and therefore negatively impacted the NUE and rice yield. Paddy *Azolla*, a good green manure, is considered as a promising measure to decrease AV and improve NUE and grain yield of rice. However, there is limited information on the integrated effects of RN and *Azolla* cover on the AV, NUE, and rice yield, especially in the highly fertilized rice-growing systems.

**Methods:**

The experiment was conducted including eight treatments: the control (without N fertilizer and *Azolla* cover), *Azolla* cover without N fertilizer (A), farmer’s N application rate (FN), FN + *Azolla* cover (FNA), 15% RN from FN (RN_15_), RN_15_ + *Azolla* cover (RN_15_A). 30% RN from FN (RN_30_), RN_30_ + *Azolla* cover (RN_30_A). The integrated effects of N fertilizer reduction and* Azolla* cover on AV, NUE, and rice grain was evaluated.

**Results:**

RN_15_A and RN_30_A substantially reduced total AV by 50.3 and 66.9% compared with FN, respectively, primarily due to the lower surface water ammonia concentrations and pH. RN improved the efficiency of *Azolla* cover on reducing AV, with 4.1–9.9% higher than for FN. Compared with the FN, RN_15_A and RN_30_A enhanced apparent N recovery efficiency (ANRE) by 46.5 and 39.1%, which might be responsible for the lower NH_3_ emission and the increased total N uptake / total chemical N applied. Furthermore, RN_15_A and RN_30_A reduced yield-scaled volatilization by 52.3 and 64.3% than for FN, respectively. Thus, combining 15–30% RN with *Azolla* cover may be a way to reduce AV and improve ANRE without decreasing rice grain yield.

## Introduction

China is the largest user of synthetic nitrogen (N) in the world (FAO, 2019). In China, the annual N consumption was accounted for approximately 30% of the world’s total N use (Huang & Zou, 2020). The average amount of chemical N fertilizer used in rice production in China was 180 kg ha^−1^ (Peng, Tang & Zou, 2009; Chen et al., 2014). In Jiangsu Province of Taihu Region, the N application rate for rice production ranged from 270 to 330 kg ha^−1^, and the average amount of N application was 300 kg ha^−1^ (Peng et al., 2011; Yao et al., 2018a). The average apparent use efficiency and agronomic use efficiency of N fertilizer of rice were 39.0% and 12.7 kg kg^−1^, respectively (Yu & Shi, 2015). Excessive N fertilizer input with low N utilization efficiency (NUE) caused a large amount of N losses (Huang et al., 2016). Ammonia volatilization (AV) from paddy fields is a principal pathway of N loss (Zhang et al., 2014). Previous researchers estimated that 10–40% of chemical N fertilizer application lost via AV (Zhang et al., 2014; Sun et al., 2015; Feng et al., 2017). A large amount of AV caused many environmental problems such as air quality degradation (Wei et al., 2015), water eutrophication, soil acidification, and biodiversity loss (Liu et al., 2013; Ti et al., 2018). Thus, it is urgent to find efficient ways to reduce the AV from paddy fields.

To reduce the AV and increase the NUE of rice, many technical measures have been developed, such as applying enhanced efficiency fertilizers (slow / controlled-release fertilizers and urease inhibitors) (Linquist et al., 2013; Wang et al., 2018; Yang et al., 2019; Yang et al., 2020), using new types of biochar (Chu et al., 2020; Yu et al., 2020), and improving fertilization techniques (split applications of N fertilizer and N fertilizer deep placement) (Huang et al., 2016; Liu et al., 2015; Yao et al., 2018a). However, enhanced efficiency fertilizers and biochar are generally too expensive, and thereby, limiting their use in rice production. In addition, their effectiveness in migrating AV, and improving NUE and rice yield was influenced by many factors such as climate conditions, soil type, and agronomic measures (Linquist et al., 2013; Abalos et al., 2014; Silva et al., 2016; Sha et al., 2019). The improved fertilization techniques need more labor or knowledge required in N fertilizer management, and thus, they are not commonly adopted by farmers (Yao et al., 2018b; Huang & Zou, 2020).

Reducing the amount of N fertilizer of rice has been recommended as a feasible way to reduce AV, increase NUE, and maintain rice yield in a highly fertilized rice-growing system (Deng et al., 2012; Qiao et al., 2012; Guo et al., 2019). Yu, Xue & Yang (2015) reported that a 22–44% reduction of N fertilizer application led to a 20–35% AV reduction from paddy fields. A meta-analysis suggested that ≤25% reduction of N fertilizer application rate improved partial factor productivity of N fertilizer and rice yield by 25% and 6.3%, respectively, in a highly fertilized rice-growing system (Guo et al., 2019). However, continuous N fertilizer reduction led to a nutrient deficiency in the soil and therefore negatively impacted the NUE and rice yield (Guo et al., 2019).

Previous studies reported that N fertilizer reduction together with green manure was more efficient in improving NUE and stabling rice grain yield compared with N fertilizer reduction alone (Guo et al., 2019; Zhang et al., 2020). *Azolla*, a floating pteridophyte, is commonly grown as an intercrop with rice and used as a green manure after death (Kollah, Patra & Mohanty, 2015). It forms a mat that covers the surface water to reduce AV by preventing the escape of NH_3._ It could also absorb the NH_4_^+^ and decrease the temperature and pH of the surface water, thereby reducing AV (Yao et al., 2018c; Yang et al., 2020). As green manure, *Azolla* can enhance the properties of the soil and enhance the microbial population of the soil, thereby improving soil fertility and rice yield (Kollah, Patra & Mohanty, 2015; Subedi & Shrestha, 2015; Yao et al., 2018c).

Previous studies have suggested that the integrated use of N fertilizer reduction and *Azolla* cover markedly reduced AV and improved NUE compared with conventional N application rate (De Macale & Vlek, 2004; Kern & Vlek, 2007; Yao et al., 2018c). For most of the previous studies, the integrated effect of N fertilizer reduction and *Azolla* cover was investigated under the low quantity of N application (<160 kg N ha^−1^) (De Macale & Vlek, 2004; Kern & Vlek, 2007). There is rarely study on the interaction influence of N fertilizer reduction and *Azolla* cover on AV, NUE, and grain yield under the highly fertilized rice-growing systems. Previous studies observed that high concentrations of ammonium N (c(NH_4_^+^)) in the surface water depressed the growth and N absorption as well as N fixation of *Azolla* (Costa et al., 2009; Yao et al., 2018c). Whether combining N fertilizer reduction and *Azolla* cover further exhibits beneficial effects in decreasing the AV, and enhancing the NUE and rice yield in highly fertilized rice-cropping systems is needed to investigate and confirm.

Therefore, this study investigated the integrated influence of N fertilizer reduction and *Azolla* cover on c(NH_4_^+^), pH, and AV in the surface water. Also, we investigated their integrated impact on rice yield, total biomass, N uptake and utilization. The aims of our study were to (1) comprehensively investigate the influence of N fertilizer reduction and *Azolla* cover on AV, NUE, and rice grain yield; (2) determine a proper percentage of N fertilizer reduction aiming to achieve a balance between rice production and environmental impacts in the *Azolla*-covered paddy fields.

## Materials and Methods

### Experimental design

A pot experiment was carried out in Bai Ma Experimental Station (31°36′N, 119°11′E), located in Taihu Region, China. The length, width, and height of the pot used in this study was 25, 20, and 35 cm, respectively. The soil which was taken from a nearby paddy field was classified as Hydragric Anthrosolos. Table 1 shows the initial properties of the soil. The experiment involved four N fertilizer application rates and two *Azolla* treatments (eight treatments in total). The eight experimental treatments contained the control (without N fertilizer and *Azolla* cover), *Azolla* cover without N fertilizer (A), farmer’s N application rate (FN, 330 kg ha^−1^), FN + *Azolla* cover (FNA), 15% N reduction from FN (RN_15_), RN_15_ + *Azolla* cover (RN_15_A), 30% N reduction from FN (RN_30_), RN_30_ + *Azolla* cover (RN_30_A). The pot experiment was performed with four replicates.

**Table 1:** The initial properties of the soil.

Soil properties	Organic matter (g kg^−1^)	Total nitrogen (N) (g kg^−1^)	Total phosphorus (P) (g kg^−1^)	Available N (mg N kg^−1^)	Available P (mg P_2_0_5_ kg^−1^)	Available potassium (mg K_2_0 kg^−1^)
Values	24.60	0.90	0.24	150.41	11.85	96.32

The experimental variety was Nanjing 9108, which was classified as a late-maturing medium japonica variety. This variety was suitable for growing in the central and southern regions of Jiangsu Province. The growth duration and grain yield of this variety were approximately 149–153 d and 9200–10000 kg ha^−1^ (Wang et al., 2013). The rice seedlings were transplanted into the plastic pots with a density of 2 hills per pot on June 28 in 2019. For each fertilized treatment, 40% of chemical N applied was broadcast on the surface water at basal fertilization period (the same date as transplanting). 20% of the chemical N applied was broadcast at tillering fertilization period (ten days after transplanting), and 40% of the chemical N applied was broadcast at panicle fertilization period (thirty-eight days after transplanting). The potash (K) fertilizer was potassium chloride (K_2_O = 60%) and phosphate (P) fertilizer was superphosphate (P_2_O_5_ = 16%). The amount of K fertilizer application was 112.5 kg K_2_O ha^−1^, and the amount of P fertilizer application was 67.5 kg P_2_O_5_ ha^−1^. 50% of the total K fertilizer applied was broadcast on the surface water at basal fertilization period, and the remaining 50% of total K fertilizer applied was broadcast at panicle fertilization period. All the P fertilizer was broadcast on the surface water at basal fertilization period. A shallow water layer (3–5 cm) was maintained in the pots before the grain-filling stage. During the grain-filling stage, alternating wetting and drying (AWD) were used. The pots were drained at 10 d before maturity. 200 g m^−2^ of fresh *Azolla* (*Azolla filiculoides Lamarck*) was applied into the pots one day before basal fertilizer application. The initial coverage of the surface water was approximately 20%. *Azolla* covered almost all the surface water 5–7 days after inoculation. The *Azolla* grew naturally on the surface water before the rice filling stage. Most of *Azolla* died off because of the AWD irrigation during the rice filling stage.

### Sampling and measurements

#### Ammonia volatilization

The ventilation method was used to measure ammonia volatilization (AV) (Xu et al., 2012; Sun et al., 2019a). After soaking with phosphoglycerol, two sponges (diameter = 15 cm and thickness = two cm) were laid in a PVC pipe (diameter = 15 cm and height = 20 cm) to collect the NH_3_. The upper sponge was placed at the top of the PVC pipe, and the lower sponge was placed three cm below the upper sponge. After each N application, the PVC pipe with two sponges was inserted into the soil. The samples in the lower sponge were collected daily in the first three days after N application and then were collected every two days. The samples were continuously collected for 7–10 days. And then, the samples were extracted with 300 mL of 1.0 mol L^−1^ KCl solution into a bottle (500 mL) and shaken for 1 h at 25 °C. The AA III continuous flowing analytical system (Bran + Luebbe company, Germany) was used to determine the c(NH_4_^+^). The ammonia volatilization flux (*AVF*, kg N ha^−1^ d^−1^) and cumulative ammonia volatilization (*CAV*, kg N ha^−1^) were calculated as follows:

\begin{eqnarray*}\mathit{AV F}= \frac{\mathit{M}}{\mathit{A}\times \mathit{T}} \times 1{0}^{-2} \end{eqnarray*}

*CAV* = ∑*AV F*

*M* refers to the quantity of NH_3_ (mg) absorbed by the phosphoglycerol-soaked sponges. *A* refers to the cross-sectional area (m^2^) of the sponges. *T* refers to the sampling interval (d).

#### Surface water c (NH_4_^+^) and pH value

100 mL surface water was sampled from the pots to determine the c(NH_4_^+^) and pH simultaneously when the AV was measured. AA III continuous flowing analytical system was used to measure the c(NH_4_^+^) in the surface water. In addition, a portable pH meter was used to measure the surface water pH.

#### Yield-scaled NH_3_ emission and apparent N recovery efficiency (ANRE)

The yield-scaled NH_3_ emission and *ANRE* were calculated as follows:

\begin{eqnarray*}\text{Yield-scaled}{\mathrm{NH}}_{3}\text{emission}= \frac{\mathit{TAV }}{\mathit{GY }} \end{eqnarray*}

\begin{eqnarray*}ANRE= \frac{{TNU}_{N}-{TNU}_{control}}{{N}_{a}} \end{eqnarray*}

where *TAV* is the total cumulative NH_3_ emission (g N kg^−1^ grain) during the whole growing season of rice. *GY* refers to the rice grain yield under different treatments. *TNU*_*N*_ and *TNU*_*control*_ refer to the total N uptake of rice plants under the N treatments and the control at maturity, respectively. *Na* refers to the total chemical N fertilizer applied.

#### Plant sampling

After maturation, three pots of rice were destructively sampled for each treatment. The leaves, stems, and panicles of rice plants were separated and then were dried in an oven at 105 °C for 30 min and then at 80 °C. After weighing the dry matter of plant organs, the plant organs were crushed with a grinder. The samples were first digested with H_2_SO_4_ and H_2_O_2_, and then the N concentrations in these samples were determined using AA III continuous flowing analytical system. At maturity, three pots of rice were harvested to measure the grain yield (GY) and its components.

### Statistical analysis

The statistical analysis was conducted by two-way analysis of variance (ANOVA) in SPSS 20.0. The combined influence of N fertilizer reduction and *Azolla* cover on the AV, GY, total biomass, straw yield, harvest index, TNU, and ANRE were tested with a general mixed linear model at *p* < 0.05. N fertilizer reduction and *Azolla* cover were treated as fixed effects and the replicate as a random effect. The *LSD* test was applied to determine the statistical differences between means under different treatments at *p* < 0.05. Simple correlation analysis was used to determine the correlation between AV and c(NH_4_^+^), pH of the surface water.

## Results

### Ammonia volatilization fluxes and cumulative ammonia volatilization

**Figure 1:**
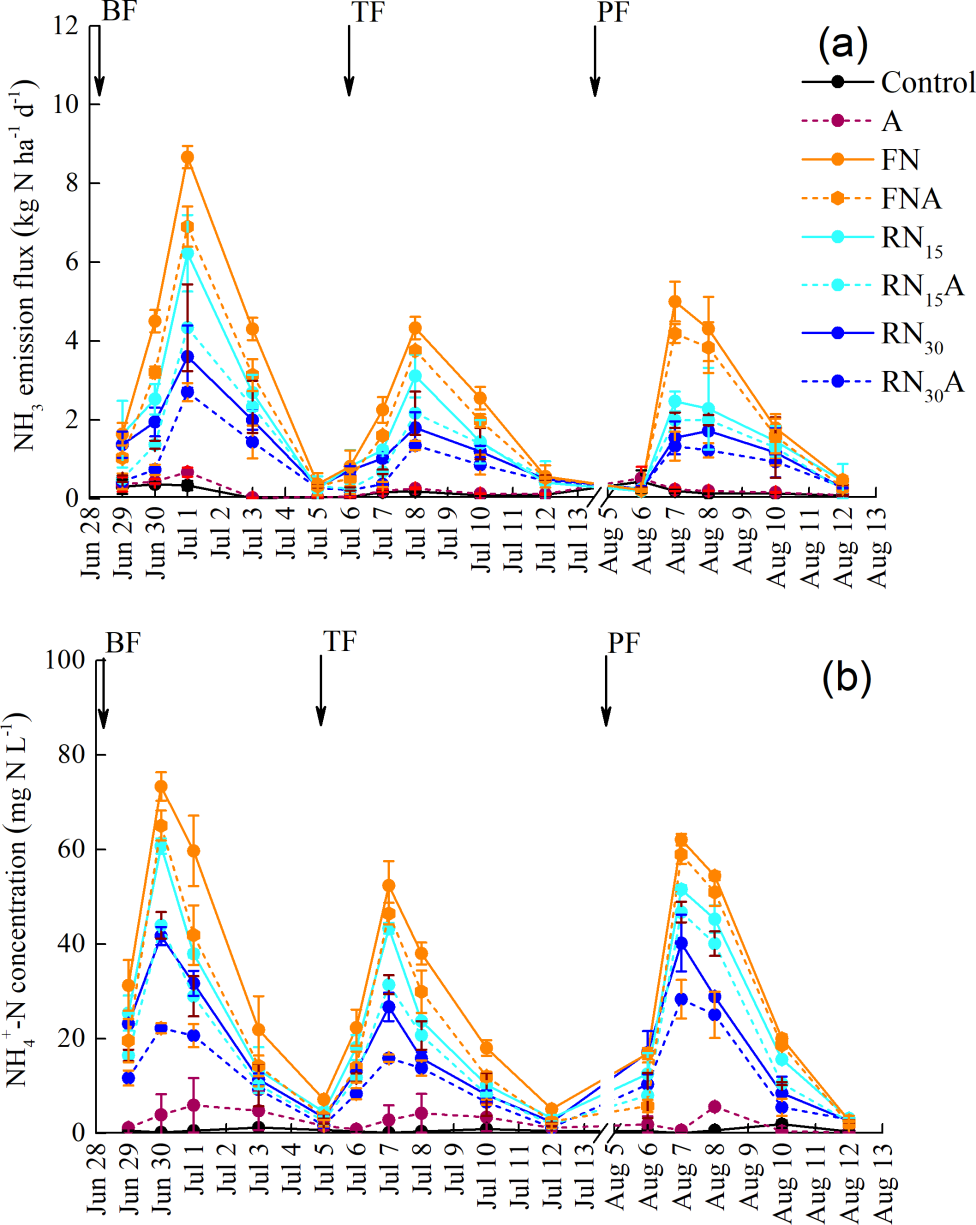
Dynamic variations of NH_3_ emission flux (A) and surface water NH${}_{4}^{+}$-N concentration (B) under different treatments. BF, basal fertilization period; TF, tillering fertilization period; PF, panicle fertilization period. Control, without N fertilizer and *Azolla* cover; A, *Azolla* cover without N fertilizer; FN, farmer’s N application rate; FNA, FN + *Azolla* cover; RN_15_, 15% N reduction from FN, RN_15_A; RN_15_ + *Azolla* cover; RN_30_, 30% N reduction from FN; RN_30_A, RN_30_ + *Azolla* cover. Vertical bars represent the standard deviations of mean (*n* = 3).

Figure 1 shows the dynamic variations of the ammonia volatilization fluxes (AVF) and NH_4_^+^-N concentrations (c(NH_4_^+^)) in the surface water after each urea application under different treatments. The maximum AVF was observed at 2–3 days after urea application, and it rapidly declined to a low level at 7 days after urea application (Fig. 1A). As expected, the peak values of AVF decreased with the decreasing N rates. The peak values of AVF after each urea application were 4.3–8.7 kg ha^−1^ d^−1^ under the FN treatment, 2.5–6.2 kg ha^−1^ d^−1^ under the RN_15_ treatment, and 1.7–3.6 kg ha^−1^ d^−1^ under the RN_30_ treatment. The *Azolla* cover resulted in a lower peak value of AVF. The decrease of the peak value of AVF ranged from 10.9–25.6% under the FNA to 12.5–33.2% under the RN_15_A, and to 24.9–38.0% under the RN_30_A. This indicated that the benefit of *Azolla* cover is more evident under N fertilizer reduction than for farmer’s N application rate. Combined N fertilizer reduction and *Azolla* cover substantially reduced the AVF*.* RN_15_A and RN_30_A reduced the AVF by 50.0–60.1% and 68.8–73.3% compared to the FN, respectively (Fig. 1A).

The cumulative NH_3_ volatilization (CAV) under the combined application of N fertilizer reduction and *Azolla* cover was presented in Table 2. The total cumulative NH_3_ volatilization (TAV) for N treatments was 17.7–51.9 kg N ha^−1^, accounting for 7.5–15.7% of the total N application rate (Na). The CAV during the basal fertilizer stage (BF) was higher than those of during tillering and panicle fertilizer stages (TF and PF). N fertilizer reduction significantly decreased the TAV (*p* < 0.05). The highest TAV was 51.93 kg N ha^−1^ for the FN and was account for 15.74% of the total urea N applied. The TAV significantly decreased to 34.48 kg N ha^−1^ for RN_15_ and 24.91 kg N ha^−1^ for RN_30_. Compared with uncovered treatment, *Azolla* cover significantly reduced the TAV, and the magnitude varied among the N application rates as detected by a significant N × *Azolla* interaction. *Azolla* cover reduced the TAV by 21.0% under the FN, by 25.1% under the RN_15_, and by 30.9% under the RN_30_. Combining N fertilizer reduction and *Azolla* cover significantly reduced the TAV*.* The TAV under RN_15_A and RN_30_A were 50.3% and 66.9% lower than that of the FN, respectively (Table 2).

**Table 2:** The combined effect of nitrogen fertilizer reduction and *Azolla* cover on the cumulative ammonia volatilization.

Treatment	Ammonia volatilization (kg N ha^−1^)				TAV / N_a_ (%)
	BF	TF	PF	Total	
Control	1.13 ± 0.04 e	0.77 ± 0.03 f	1.21 ± 0.07 f	3.11 ± 0.06 f	–
A	1.63 ± 0.14 e	1.03 ± 0.10 f	1.48 ± 0.15 f	4.14 ± 0.23 f	–
FN	24.19 ± 3.57 a	13.66 ± 1.17 a	14.09 ± 1.58 a	51.93 ± 3.09 a	15.74
FNA	18.08 ± 1.11 b	10.93 ± 0.47 b	12.00 ± 0.96 b	41.01 ± 1.87 b	12.43
RN_15_	16.43 ± 2.33 b	9.29 ± 1.27 c	8.76 ± 0.50 c	34.48 ± 3.88 c	12.32
RN_15_A	11.54 ± 2.16 c	6.99 ± 1.22 d	7.30 ± 1.05 cd	25.83 ± 4.26 d	9.22
RN_30_	11.53 ± 0.39 c	6.94 ± 0.06 d	6.44 ± 1.25 de	24.91 ± 1.47 d	10.83
RN_30_A	7.32 ± 1.19 d	4.59 ± 0.64 e	5.30 ± 0.60 e	17.21 ± 1.74 e	7.48
Source of variance	Probability values				
Nitrogen	<0.001	<0.001	<0.001	<0.001	–
*Azolla*	<0.001	<0.001	0.01	<0.001	–
Nitrogen ×*Azolla*	0.005	0.019	0.193	0.005	–

**Notes.**

BF basal fertilization period TF tillering fertilization period PF panicle fertilization period Control without N fertilizer and Azolla cover A Azolla cover without N fertilizer FN farmer’s N application rate FNA FN + Azolla cover RN_15_ 15% N reduction from FN; RN_15_A, RN_15_ + Azolla cover RN_30_ 30% N reduction from FN RN_30_A RN_30_ + Azolla cover

*TAV*/*N*_*a*_ indicates the ratio of total cumulative ammonia volatilization to the total N fertilizer applied.

In each column, different lowercase letters indicate statistically significant difference at the 0.05 level between different treatments.

Values represent mean ± standard deviation.

### Surface water c(NH_4_^+^) and pH

In line with the AVF, the surface water c(NH_4_^+^) showed the same trend (Fig. 1B). The largest surface water c(NH_4_^+^) after each urea application ranged from 52.3 to 73.3 mg N L^−1^ under the FN, from 43.4 to 60.7 mg N L^−1^ under the RN_15_, and from 26.7 to 41.7 mg N L^−1^ under the RN_30_ (Fig. 1B). Compared with uncovered treatment, *Azolla* cover significantly decreased the c(NH_4_^+^) by 5.0–11.3% under the FN, by 9.3–27.6% under the RN_15_, and by 29.5–46.7% under the RN_30_ (Fig. 1B). This indicated that the efficiency of *Azolla* cover in decreasing c(NH_4_^+^) in surface water under N fertilizer reduction was higher than that of farmer’s N application rate. Combining N fertilizer reduction and *Azolla* cover decreased the surface water c(NH_4_^+^) substantially*.* Compared with the FN, RN_15_A and RN_30_A reduced the c(NH_4_^+^) in surface water by 24.7–40.0% and 54.4–70.0%, respectively (Fig. 1B).

Compared with the control, N treatment increased the surface water pH during the BF and TF. The average surface water pH was reduced by 0–0.1 units under the RN_30_ compared with the FN. The impact of RN_15_ on the pH of surface water was smaller compared with the FN. Compared with uncovered treatment, *Azolla* cover reduced the surface water pH, and the efficiency of *Azolla* on reducing surface water pH varied under different N application rates. *Azolla* cover decreased the surface water pH 0–0.1 units under the RN_30_, while the effects of *Azolla* cover under the RN_15_ and FN on surface water pH were relatively small. Moreover, compared with the FN, RN_15_A, and RN_30_A reduced the pH value of surface water 0–0.1 and 0.1–0.3 units, respectively (Table 3).

**Table 3:** The combined effect of nitrogen fertilizer reduction and Azolla cover on the surface water pH.

Treatment	BF			TF			PF	
	Range	Mean ± SD		Range	Mean ± SD		Range	Mean ± SD
Control	7.34–8.10	7.75 ± 0.28		7.34–7.85	7.63 ± 0.19		7.52–8.10	7.86 ± 0.24
A	7.11–7.74	7.55 ± 0.20		7.11–7.71	7.55 ± 0.25		7.49–8.06	7.74 ± 0.22
FN	7.59–8.47	8.18 ± 0.35		7.79–8.47	8.11 ± 0.33		7.61–8.58	8.12 ± 0.44
FNA	8.03–8.32	8.13 ± 0.11		7.90–8.32	8.09 ± 0.16		7.71–8.58	8.09 ± 0.38
RN_15_	7.91–8.45	8.15 ± 0.39		7.85–8.25	8.08 ± 0.25		7.47–8.45	7.95 ± 0.41
RN_15_A	7.97–8.33	8.13 ± 0.13		7.70–8.17	8.01 ± 0.15		7.31–8.33	7.99 ± 0.42
RN_30_	7.73–8.51	8.12 ± 0.26		7.48–8.51	7.96 ± 0.36		7.78–8.27	8.01 ± 0.23
RN_30_A	7.73–8.44	8.01 ± 0.28		7.42–8.01	7.80 ± 0.15		7.60–8.62	8.04 ± 0.46

**Notes.**

BF basal fertilization period TF tillering fertilization period PF panicle fertilization period Control without N fertilizer and Azolla cover A Azolla cover without N fertilizer FN farmer’s N application rate FNA FN + Azolla cover RN_15_ 15% N reduction from FN RN_15_A RN_15_ + Azolla cover RN_30_ 30% N reduction from FN RN_30_A RN_30_ + Azolla cover

SD represents the standard deviation of mean (*n* = 5).

**Figure 2:**
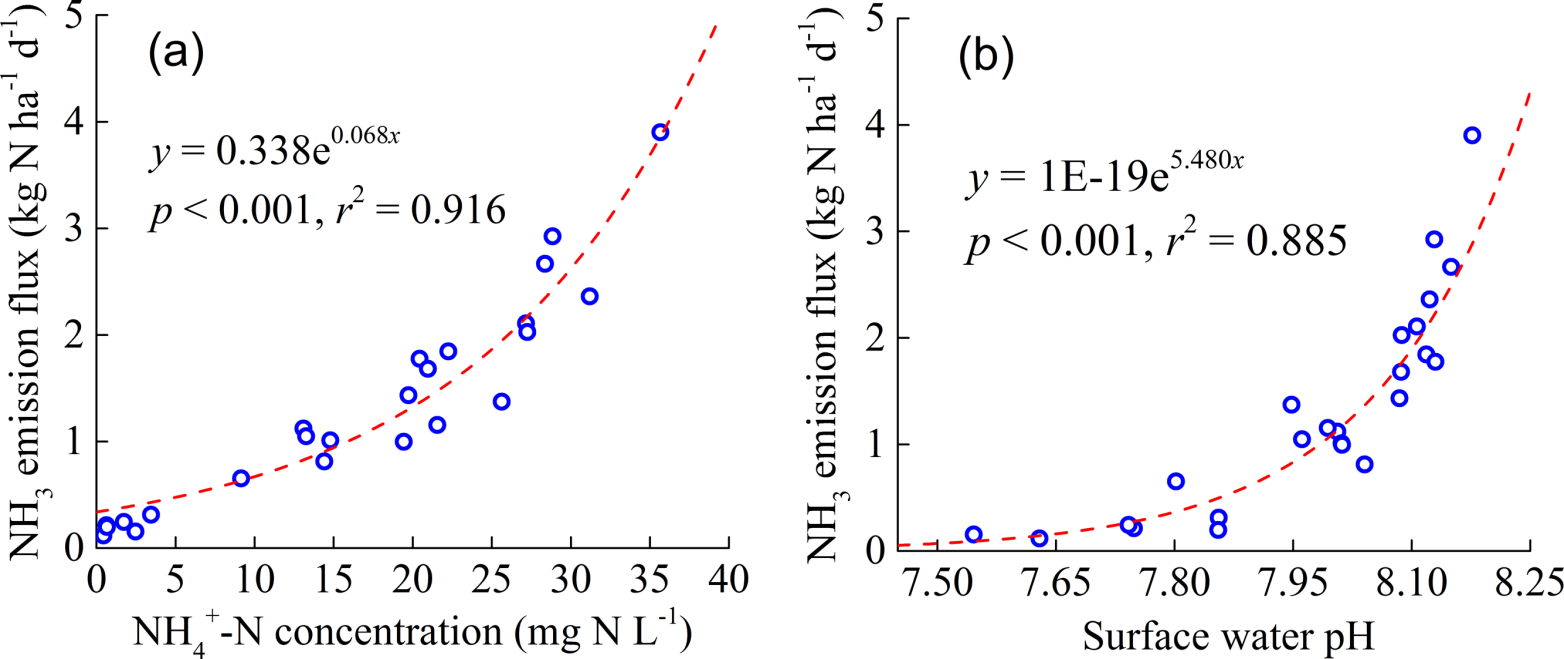
The relationship between ammonia (NH_3_) emission flux and surface water NH${}_{4}^{+}$-N concentration (A) and surface water pH (B).

The relationships between AVF, c(NH_4_^+^), and pH in the surface water were well described with the exponential models (*p* < 0.001) (Fig. 2). At low c(NH_4_^+^) and pH of surface water, the AVF increased slowly as both parameters increased. However, at high c(NH_4_^+^) and pH in the surface water, the AVF increased sharply with the increase of two parameters (Fig. 2).

### Rice yield, total biomass, straw yield, and harvest index

The impacts of N fertilizer reduction on the grain yield (GY) were significant (*p* < 0.05), whereas the effect of *Azolla* cover and the interaction effect of N fertilizer reduction and *Azolla* cover on GY were not significant (Table 4). The GY for the RN_30_ treatment was 70.04 g pot^−1^, and the GY significantly increased to 80.01 g pot^−1^ for RN_15_ treatment and 81.26 g pot^−1^ for FN treatment. Despite the absence of statistical differences, compared with uncovered treatment, *Azolla* cover increased the GY by 2.9% under the FN, by 6.3% under the RN_15_, and by 7.7% under the RN_30_, respectively. It demonstrated that the positive effect of *Azolla* cover on the GY was pronounced under N fertilizer reduction than under farmer’s N application rate. In addition, we observed that rice yield for RN_30_A was comparable to for the FN, and a higher rice yield was obtained for RN_15_A than for FN (Table 4).

**Table 4:** The combined effect of nitrogen fertilizer reduction and Azolla cover on the grain yield, total biomass, straw yield and harvest index.

Treatment	Grain yield (g pot^−1^)	Total biomass (g pot^−1^)	Straw yield (g pot^−1^)	Harvest index
Control	56.81 ± 2.86 d	95.91 ± 7.26 d	39.10 ± 8.90 d	0.60 ± 0.06 a
A	62.65 ± 5.46 d	104.64 ± 12.10 d	41.99 ± 7.41 cd	0.59 ± 0.01 ab
FN	81.26 ± 6.85 ab	165.53 ± 10.16 ab	84.28 ± 8.56 ab	0.49 ± 0.04 c
FNA	83.63 ± 5.17 a	178.32 ± 9.87 a	94.68 ± 6.51 a	0.47 ± 0.08 c
RN_15_	80.10 ± 5.12 ab	157.40 ± 3.97 b	77.30 ± 9.09 b	0.52 ± 0.10 bc
RN_15_A	85.17 ± 7.37 a	175.58 ± 6.07 a	90.41 ± 6.64 ab	0.49 ± 0.08 c
RN_30_	70.04 ± 6.85 c	124.03 ± 10.58 c	53.99 ± 7.58 c	0.57 ± 0.07 ab
RN_30_A	75.42 ± 5.04 bc	157.89 ± 7.85 b	82.47 ± 7.35 ab	0.48 ± 0.05 c
Source of variance	Probability values			
Nitrogen	<0.001	<0.001	<0.001	0.002
*Azolla*	0.05	<0.001	0.001	0.051
Nitrogen × *Azolla*	0.97	0.11	0.07	0.33

**Notes.**

Control without N fertilizer and Azolla cover A Azolla cover without N fertilizer FN farmer’s N application rate FNA FN + Azolla cover RN_15_ 15% N reduction from FN RN_15_A RN_15_ + Azolla cover RN_30_ 30% N reduction from FN RN_30_A RN_30_ + Azolla cover

In each column, different lowercase letters indicate statistically significant difference at the 0.05 level between different treatments.

Values represent mean standard deviation (*n* = 3).

The effects of N fertilizer reduction on total biomass (TB), straw yield (SY), and harvest index (HI) were significant (*p* < 0.05) (Table 4). The TB and SY decreased with the decreasing N fertilizer application rate. However, the HI increased with the decreasing N fertilizer application rate. The *Azolla* cover had a significant effect on the TB and SY, but it had no significant effect on the HI. There was no significant difference in TB, SY, and HI between RN_15_A, RN_30_A, and FN (Table 4).

### N absorption and use efficiency

**Figure 3:**
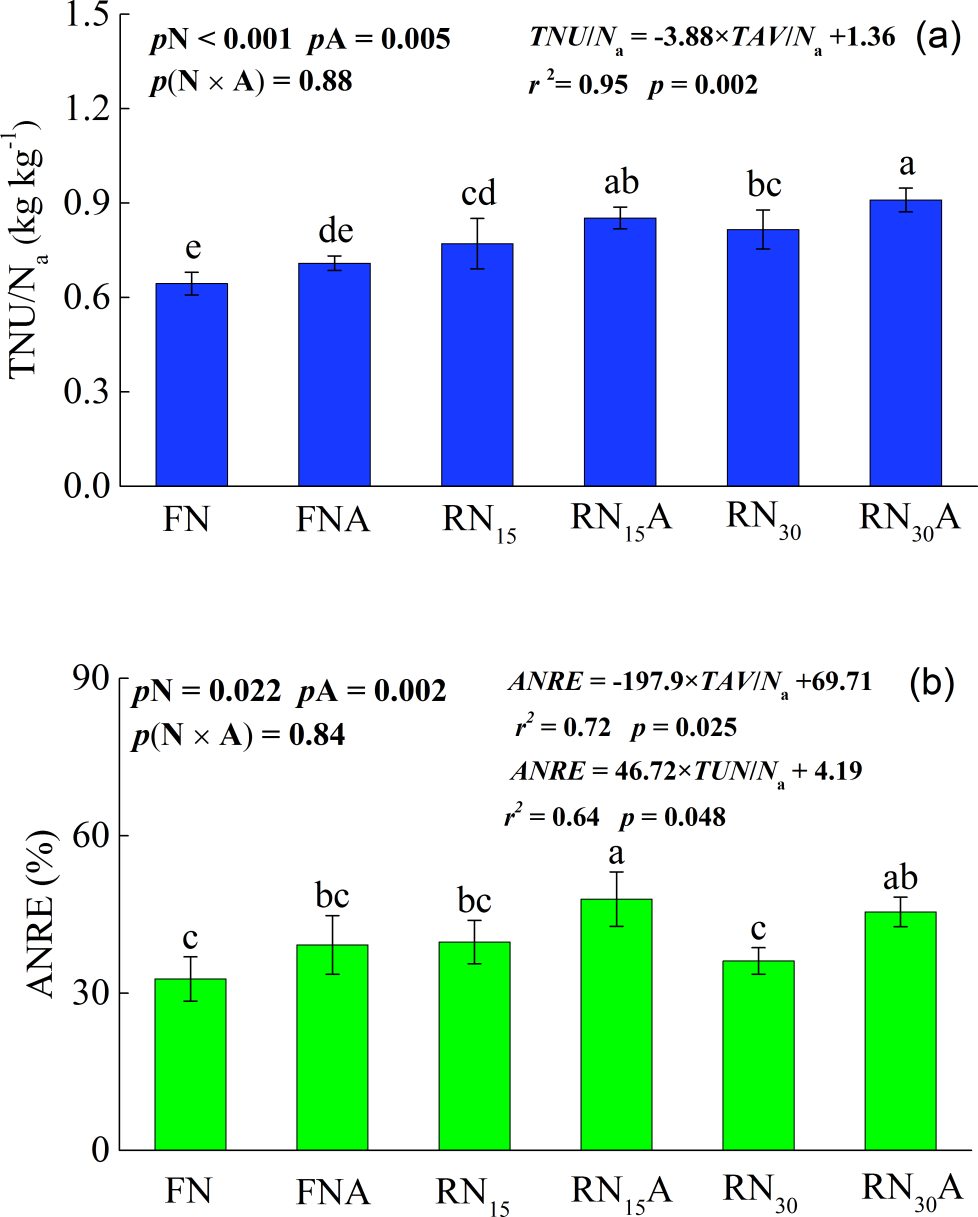
The total nitrogen uptake/total chemical N applied (TNU/N_*a*_) (A) and apparent nitrogen recovery efficiency (ANRE) (B) under different treatments. Control, without N fertilizer and *Azolla* cover; A, *Azolla* cover without N fertilizer; FN, farmer’s N application rate; FNA, FN + *Azolla* cover; RN_15_, 15% N reduction from FN; RN_15_A, RN_15_ + *Azolla* cover; RN_30_, 30% N reduction from FN; RN_30_A, RN_30_ + *Azolla* cover. *p*N, *p*A, and *p* (N × A) represent the probability values of the effect of nitrogen fertilizer treatment, *Azolla* treatment and their interaction. *TAV*/*N*_*a*_ indicates the ratio of total cumulative ammonia volatilization to the total N fertilizer applied. Vertical bars represent the standard deviations of the mean (*n* = 3).

Figure 3 shows the integrated effect of N fertilizer reduction and *Azolla* cover on the total N uptake / total chemical N applied (TNU / N_a_) and apparent N recovery efficiency (ANRE). The influence of N fertilizer reduction and *Azolla* cover on the TNU / N_a_ and ANRE was significant (*p* <0.05), but their interaction effect on the TNU / N_a_ and ANRE was not significant. The ANRE under different N treatment were in the order of RN_15_ >RN_30_ >FN. This indicated that an appropriate N fertilizer reduction could obtain a higher ANRE. The combined application of N fertilizer reduction and *Azolla* cover led to the higher TNU / N_a_ and ANRE than N fertilizer reduction alone. However, the magnitude of increase in TNU / N_a_ and ANRE by applying *Azolla* cover varied under different N application rates. Compared with N fertilizer reduction, *Azoll* a cover increased TNU / N_a_ and ANRE by 10.1 and 19.9% for FNA, by 10.6% and 20.6% for RN_15_A, and by 11.5 and 25.9% for RN_30_A, respectively. Moreover, compared with the FN treatment, RN_15_A and RN_30_A significantly increased ANRE by 46.5 and 39.1%, respectively (Fig. 3). The correlation analysis showed that ANRE was negatively correlated with TAV / Na (*r*^2^ = 0.72) and was positively correlated with TNU / Na (*r*^2^ = 0.64). Thus, the higher ANRE might be attributed to the reduced TAV / Na and the enhanced TNU / Na.

### Yield-scaled NH_3_ volatilization

**Figure 4:**
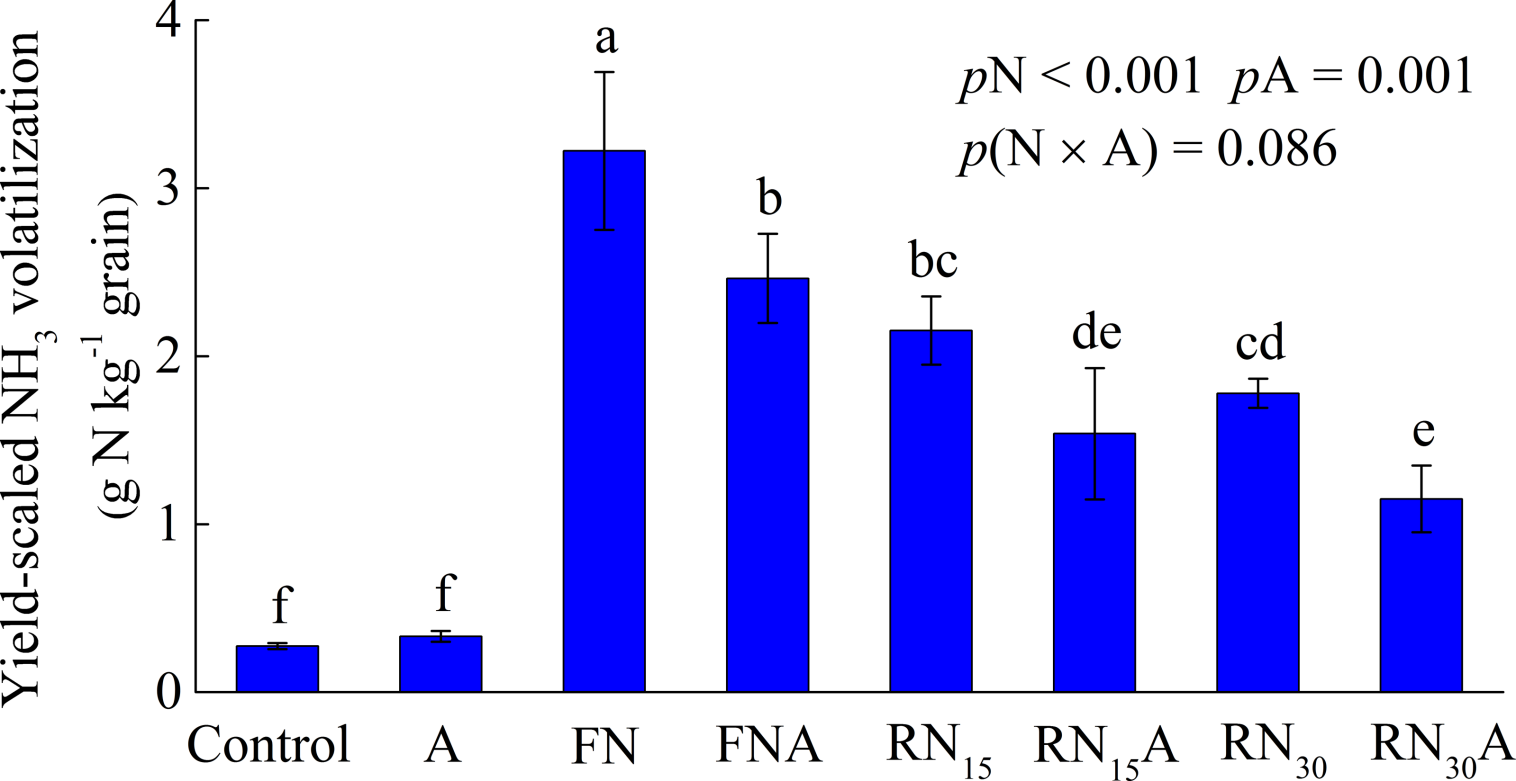
The yield-scaled NH_3_ volatilization under different treatments. Control, without N fertilizer and *Azolla* cover; A, *Azolla* cover without N fertilizer; FN, farmer’s N application rate; FNA, FN + *Azolla* cover; RN_15_, 15% N reduction from FN; RN_15_A, RN_15_ + *Azolla* cover; RN_30_, 30% N reduction from FN; RN_30_A, RN_30_ + *Azolla* cover. *p*N, *p*A, and *p* (N × A) represent the probability values of the effect of nitrogen fertilizer treatment, *Azolla* treatment and their interaction. Vertical bars represent the standard deviations of the mean (*n* = 3).

Figure 4 shows the yield-scaled ammonia volatilization under different treatments. The effect of N fertilizer reduction and *Azolla* cover treatments on yield-scaled ammonia volatilization was significant (*p* < 0.05). However, their interaction effect on yield-scaled NH _3_ volatilization was not significant. The yield-scaled NH _3_ volatilization was 1.78 g N kg^−1^ grain for RN_15_, and 2.15 g N kg^−1^ grain for RN _30_. It substantially increased to 3.22 g N kg^−1^ grain for FN, which was the highest AV-producing treatment. *Azolla* cover significantly reduced yield-scaled NH _3_ volatilization by 23.6–35.3%, and the magnitude of yield-scaled NH_3_ volatilization reduction was higher for the RN_15_A and RN_30_A than for the FNA. The combined application of N fertilizer reduction and *Azolla* cover significantly decreased yield-scaled NH _3_ volatilization. RN_15_A and RN_30_A reduced yield-scaled NH_3_ volatilization by 52.3 and 64.3% compared with the FN, respectively.

## Discussion

### Combined effect of N fertilizer reduction and Azolla cover on NH_3_ emission

In this study, the TAV under different treatments was responsible for 7.5–15.7% of the total N applied (Table 2). Similar results were observed by other previous studies (Cao et al., 2013; Feng et al., 2017; Sun et al., 2019b). Reducing N application was one of the primary strategies to achieve lower NH_3_ loss. The NH_3_ loss decreased with the reduced N application rates (Chen et al., 2015; Huang et al., 2016; Shi et al., 2020). The TAV was 51.93 kg N ha^−1^ for FN, and it significantly decreased by 34.48 kg N ha^−1^ for RN_15_ and by 24.91 kg N ha^−1^ for RN_30_ (Table 2).

*Azolla* cover can mitigate NH_3_ emission by acting as a mat to hinder the escape of NH_3_ and reduce the light intensity in the floodwater. Thus, it could depress the rise in pH caused by algal photosynthesis and decrease the temperature in the floodwater. In addition, *Azolla* cover reduced the NH_3_ loss by assimilating NH_4_^+^-N in the floodwater (Yao et al., 2018c; Yang et al., 2020). *Azolla* cover substantially reduced the TAV by 21.0 −30.9% under the N application rate (Table 2). Moreover, the efficiency of *Azolla* cover in controlling NH_3_ emission differed among the N application rates. The efficiency of *Azolla* cover in controlling NH_3_ emission increased with the decreasing N application rate (Table 2). Previous studies reported that the influence of *Azolla* cover on decreasing NH_3_ emission under low N application rates had a higher effectiveness than for high N rates (Kern & Vlek, 2007; Yao et al., 2018c). High c(NH_4_^+^) in the surface water depressed the growth and N absorption of *Azolla*, negatively impacting the efficiency in reducing NH_3_ loss (Cissé & Vlek, 2003; Kern & Vlek, 2007; Yao et al., 2018c). Costa et al. (2009) reported that 40 mg L^−1^ c(NH_4_^+^) led to an 18 and 46% inhibition of growth and N fixation of *Azolla*, respectively. In this study, the peak value of c(NH_4_^+^) in surface water after each urea application was 26.7–41.7 kg N ha^−1^ for low N rate, but they increased to as high as 43.4–60.7 kg N ha^−1^ for moderate N rate, and 52.4–73.3 kg N ha^−1^ for high N rate (Fig. 1B). This is the main reason that *Azolla* cover is more effective in reducing NH_3_ loss under the low N rate than for the high N rate.

In addition, a high N rate promoted the growth of algae plants. The algae bloom consumed a large amount of dissolved CO_2_ in surface water and led to an increase in surface water pH (Yao et al., 2017), resulting in an increase of NH_3_ emission. Light intensity on surface water is one of the principal environmental factors determining *Azolla* growth (Sadeghi et al., 2013; Pouil et al., 2020). A high N application rate produced a higher leaf area index of rice, causing more shading to the *Azolla* plants than for a low N application rate (Singh & Singh, 1988). The enhanced shading significantly suppressed the biomass and the N fixation of *Azolla* (Sadeghi et al., 2013), which might negatively influence the efficiency in reducing NH_3_ emission.

The integrated application of N fertilizer reduction *Azolla* cover could more effectively decrease the TAV compared with single N fertilizer reduction. In the present, the combined application of N fertilizer reduction and *Azolla* cover not only led to 50.3–66.9% lower TAV than for the FN treatment but also led to 25.1–30.9% lower TAV than for the N reduction alone (Table 2). The lower TAV under the combined application of N fertilizer reduction and *Azolla* cover was mainly ascribed to the lower surface water c(NH_4_^+^) and pH (Fig. 2). In our study, the combined application of N fertilizer reduction and *Azolla* cover decreased the c(NH_4_^+^) by 24.7–70.0% (Fig. 1) and pH 0.1–0.3 units in the surface water (Table 3). The lower surface water c(NH_4_^+^) was ascribed to the lower N application rate and the absorption of NH_4_^+^ by *Azolla* (Yang et al., 2020). The N fertilizer reduction decreased the pH of surface water (Silva et al., 2016). Moreover, *Azolla* cover reduced the incoming light, and therefore depressing the photosynthesis of algae. Thus, it decreased the CO_2_ consumption by algae in the surface water, resulting in a decrease in pH (Liu et al., 2017).

### Combined effects of N fertilizer reduction and Azolla cover on grain yield

Our results showed that the rice yield under the FN treatment was slightly higher than that of the RN_15_, but was significantly higher than for the RN_30_ treatment (Table 4). This indicated a 15% N fertilizer reduction from the FN treatment could maintain rice grain yield compared with the FN treatment. However, a 30% N fertilizer reduction from the FN treatment resulted in a substantial decrease in rice grain yield.

Combined proper N fertilizer reduction and *Azolla* cover could maintain grain yield of rice compared with conventional N fertilizer application (De Macale & Vlek, 2004; Kern & Vlek, 2007; Malyan et al., 2019). Guo et al. (2019) suggested that the rice yield could be improved by reducing N application when controlling the reduction of N fertilizer within 31% and combined with optimizing management. Malyan et al. (2019) reported that combined application of *Azolla* and 30% N fertilizer reduction from recommended N fertilizer led to a comparable yield with the recommended N fertilizer (120 kg N ha^−1^). We observed that the combined application of a 15–30% N fertilizer reduction from the FN treatment with *Azolla* cover produced comparable or higher rice grain yield than FN treatment (Table 4). This indicated that N fertilizer application rate could be reduced by 50–100 kg N ha^−1^ in *Azolla*-covered paddy field without significantly decreasing the grain yield. Previous study reported that 50% of the applied chemical N fertilizer was absorbed by *Azolla* during early stage, leading to the decrease of NH_3_ emission (Kollah, Patra & Mohanty, 2015). The N absorbed by *Azolla* during early stage had little impact on the growth of rice due to the lower N demand for rice seedlings (Li et al., 2018). In addition, *Azolla* could fix approximately 44–56 kg N ha^−1^ during the whole growing season (Yao et al., 2018c). Duo to the *Azolla* death and decomposition during mid-growing stage, *Azolla*-N was released and a considerable quantity of available N was absorbed by rice, and therefore, promoting the dry matter accumulation and grain yield increase (Yao et al., 2018c). Thus, there was no significant decrease in grain yield even when the N fertilizer was reduced by 100 kg N ha^−1^ in our study.

The continuous single N fertilizer reduction might result in a nutrient deficiency in the soil, and therefore increasing the risk of reduction in N use efficiency and yield (Guo et al., 2019). *Azolla* can increase the nutrient of soil, enhance the properties of the soil, and increase the microbial population of the soil (Kollah, Patra & Mohanty, 2015; Subedi & Shrestha, 2015; Teimour et al., 2018). Thus, combining N fertilizer reduction and *Azolla* cover might stable rice grain yield in the long term (Xue, Yu & Yang, 2014; Guo et al., 2019; Zhang et al., 2020).

The positive effect of *Azolla* cover on rice grain yield was more significant under the RN_30_ than under the RN _15_ and FN treatments. This result can be explained by the fact that N becomes the limiting factor in crop yield under low N rates. The N saved from NH_3_ loss duo to *Azolla* cover under low N rate greatly influenced rice grain yield (Silva et al., 2016). On the contrary, under the moderate and high N rates, the N level of the soil is relatively high, and conserving N by decreasing NH _3_ loss may not effectively be used to improve grain yield of rice (Li et al., 2015).

### Combined effects of N fertilizer reduction and Azolla cover on N absorption and use efficiency

Appropriate N fertilizer reduction improved grain yield and N absorption of rice, and therefore resulted in increased NUE (Guo et al., 2019). RN_15_ improved the ANRE compared with the FN treatment. However, RN_30_ slightly increased the ANRE than for the FN treatment (Fig. 3B). Similarly, Liu et al. (2016) suggested that 10% N fertilizer reduction from farmer’s N application rate could increase the ANRE. However, a 20–30% N fertilizer reduction from farmer’s N application rate decreased ANRE (Liu et al., 2016). In this study, RN_30_A significantly increased ANRE compared with the FN treatment (Fig. 3B). The combined application of 30% N fertilizer reduction and *Azolla* cover significantly decreased NH_3_ loss (Table 2) and enhanced N uptake of rice plants (Fig. 3A), therefore increasing the ANRE. In addition, the magnitude of increase in ANRE duo to *Azolla* cover was higher under N fertilizer reduction than that of farmer’s N fertilizer application rate (Fig. 3B). This might be attributed to the higher efficiency in reducing NH_3_ losses under the N fertilizer reduction than for farmer’s N fertilizer application rate (Table 2). The higher reduction in ammonia loss under a low N rate supplied a relatively larger quantity of N for rice than a high N rate, and therefore produced a higher N use efficiency (Yang et al., 2020; Yao et al., 2018c).

## Conclusion

This study assessed the integrated influences of nitrogen fertilizer reduction and *Azolla* cover on NH_3_ loss, nitrogen use efficiency, and rice yield. Our results showed that a 15–30% nitrogen fertilizer reduction with *Azolla* cover could significantly reduce NH_3_ emission and enhance apparent nitrogen recovery efficiency (ANRE) without decreasing rice yield. Combining nitrogen fertilizer reduction with *Azolla* cover decreased NH_4_^+^ concentrations of the surface water and pH, with subsequence reducing the NH_3_ volatilization, thereby enhancing the ANRE of rice. Overall, this study could provide an approach to reduce NH_3_ emission and improve agronomic performance in environment-friendly rice production. However, our results were supported by a pot experiment. It was not clear and needed to be studied further under field conditions.

##  Supplemental Information

10.7717/peerj.11077/supp-1 Supplemental Information 1 Raw data

## Additional Information and Declarations

### Data Availability

The following information was supplied regarding data availability: Raw data are available as a Supplemental File.
